# Children struggle beyond preschool-age in a continuous version of the ambiguous figures task

**DOI:** 10.1007/s00426-019-01278-z

**Published:** 2019-12-19

**Authors:** Eva Rafetseder, Sarah Schuster, Stefan Hawelka, Martin Doherty, Britt Anderson, James Danckert, Elisabeth Stöttinger

**Affiliations:** 1 Division of Psychology, Faculty of Natural Sciences, University of Stirling, Stirling, FK9 4LA UK; 2 Department of Psychology, Centre for Cognitive Neuroscience, University of Salzburg, Hellbrunnerstrasse 34, 5020 Salzburg, Austria; 3 School of Psychology, University of East Anglia, Norwich, NR4 7TJ UK; 4 Department of Psychology and Centre for Theoretical Neuroscience, University of Waterloo, Waterloo, ON N2L 3G1 Canada; 5 Department of Psychology, University of Waterloo, Waterloo, ON N2L 3G1 Canada

## Abstract

Children until the age of five are only able to reverse an ambiguous figure when they are informed about the second interpretation. In two experiments, we examined whether children’s difficulties would extend to a continuous version of the ambiguous figures task. Children (Experiment 1: 66 3- to 5-year olds; Experiment 2: 54 4- to 9-year olds) and adult controls saw line drawings of animals gradually morph—through well-known ambiguous figures—into other animals. Results show a relatively late developing ability to recognize the target animal, with difficulties extending beyond preschool-age. This delay can neither be explained with improvements in theory of mind, inhibitory control, nor individual differences in eye movements. Even the best achieving children only started to approach adult level performance at the age of 9, suggesting a fundamentally different processing style in children and adults.

**Electronic supplementary material:**

The online version of this article (10.1007/s00426-019-01278-z) contains supplementary material, which is available to authorized users.

## Introduction

Reversible or ambiguous figures like the Rubin’s face/vase picture or the Necker cube have been used to study how people spontaneously alternate between two mutually exclusive interpretations of objectively stable pictures. The ability to reverse ambiguous figures depends on a combination of top–down and bottom–up processes (Intaitė, Noreika, Šoliūnas, & Falter, 2013; Long & Toppino, 2004), including recurring neural fatigue (review in Long & Toppino, 1981, 2004), gaze orientation (Ruggieri & Fernandez, 1994), mental imagery (Doherty & Wimmer, 2005) and context effects (Intaitė et al., 2013). A critical factor that determines whether participants are able to reverse an ambiguous figure is the amount of information given about the two potential interpretations (Mitroff, Sobel, & Gopnik, 2006). (1) When no information is given, one needs to be aware of the ambiguity (i.e., uninformed reversal). Adults rarely ever reverse spontaneously (Rock & Mitchener, 1992). (2) When aware of the ambiguity, one needs to explore or generate potential alternative interpretations (i.e., ambiguity-informed reversal). Under these conditions, about 50% of adults are able to recognize the second interpretation (Girgus, Rock, & Egatz, 1977). (3) When both ambiguity and content are known, the perceiver must be able to conceive of a figure having more than one interpretation to flexibly alternate between those two (i.e., content informed reversal), a requirement that was only initially evident in children (Doherty & Wimmer, 2005; Gopnik & Rosati, 2001).

Children up to 5 years almost never show uninformed reversals (Girgus et al., 1977; Rock, Gopnik, & Hall, 1994; Rock & Mitchener, 1992)—and even struggle with content informed reversals (Gopnik & Rosati, 2001; Rock, Hall, & Davis, 1994; Wimmer & Doherty, 2011). In fact, even the majority of 5- to 9-year olds fail to reverse ambiguous figures spontaneously (Mitroff et al., 2006). Although reversal rates increase throughout childhood (Holt & Matson, 1976), they still do not reach adult level by age 10 (Ehlers, Strüber, & Basar-Eroglu, 2016).

This developmental phenomenon is compelling and requires further investigation for two reasons. A late onset may either reflect an ontogenetically emerging basic, low-level perceptual process or a cognitively demanding, late-manifesting process. Previous research has produced mixed findings. Gopnik and Rosati (2001) showed that reversals were closely linked with tasks that elicited non-perceptual, multiple representations (e.g., false belief task), suggesting that the ability to reverse depends upon late-developing cognitive abilities. Yet, more recent evidence (Doherty & Wimmer, 2005; Wimmer & Doherty, 2011) does not find a direct relation between false belief performance and switching interpretations. The current set of studies was designed to further investigate this question.

Another reason to investigate this phenomenon developmentally is that children, even when informed about the ambiguity, are unlikely to reverse. Ambiguity-informed reversals explicitly require children to explore or generate potential alternative interpretations. Children, however, fail to switch between interpretations—even when they have acknowledged their existence beforehand (Doherty & Wimmer, 2005). While children may simply be reluctant to switch interpretations in view of physically unchanged objects, this seems unlikely to explain their difficulties, given that children were encouraged to report any changes they saw (Gopnik & Rosati, 2001). Alternatively, once children understand that two interpretations are possible, they may struggle to (voluntarily) inhibit one interpretation over the other. An increased ability to reverse would then be an indicator of bottom–up development through successful inhibition (Bialystok & Shapero, 2005; Wimmer & Doherty, 2011).

Also, earlier studies reported an association between content informed reversal and theory of mind (Gopnik & Rosati, 2001; Mitroff et al., 2006) which was not confirmed in later studies (Doherty & Wimmer, 2005; Wimmer & Doherty, 2011). Studies that found a positive association argued that considering someone else’s belief and understanding ambiguity both require children to have an abstract understanding of perspective (for a discussion see Perner, Stummer, Sprung, & Doherty, 2002).

In the current study, we used a continuous version of the ambiguous figures task. In this task, an animal (e.g., a duck) morphs over 15 iterations into another animal (e.g., a rabbit) with picture #8 depicting a well-known ambiguous figure (e.g., duck–rabbit, Wittgenstein, 1953). This picture morphing task (Stöttinger et al., 2014; see also Burnett & Jellema, 2013) measures how many morphs participants need before they switch to the new interpretation (i.e., rabbit). Unlike in previous studies, children were not informed about the content of the second interpretation, only about its potential for another interpretation. Participants have to generate alternative perspectives (i.e., it could transform into a cat, a dog, a fish, etc.), and compare or rank perspectives (i.e., at some point it cannot be a fish anymore, given the ears) to report the target animal. Thus, possible alternatives cannot be chosen randomly but need to be consistent with the actual stimulus. Otherwise, the amount of potential alternative explanations would be overwhelming, ultimately hampering efficient updating.

The picture morphing task, therefore, provides several advantages over the standard ambiguous figure task: (1) the continuous measure makes it possible to test children younger than 5 years, who would have otherwise rarely reversed (Gopnik & Rosati, 2001). (2) It introduces variance into a typically dichotomous measure. (3) Most importantly, it allows to capture gradual developmental improvements and to quantify any specific deficits in this form of reversal.

In Experiment 1, we assessed children’s developmental progression on the picture morphing task and compared it to performance of adult participants exposed to the same stimulus material. We were particularly interested in whether younger children would need more morphing stages until they named the emerging animal compared to older children and adults. This task has never been administered in children before. It is therefore difficult to make any definitive predictions about how they will perform. In a similar vein, we can only speculate which other factors may influence recognition of the second object. We tested whether children who master the standard false belief task (Wimmer & Perner, 1983) will identify the second object in the picture morphing task sooner. In addition, we tested whether executive functioning and selective attention would influence children’s performance in the picture morphing task—both of which were previously hypothesized to affect performance on the ambiguous figures task. Increased inhibitory control (i.e., the ability to withhold the previous representation of the stimulus and exchange it for the conflicting option) could help children to ascribe a different meaning to the picture (Bialystok & Shapero, 2005). Wimmer and Doherty (2011) found that performance on the day/night Stroop task (Gerstadt, Hong, & Diamond, 1994), a measure of inhibitory control, predicted ambiguous figure switching. We, therefore, used the same task in our study.

For exploratory purposes, we also recorded children’s eye movements to test whether picture morphing effects relate to individual differences in visual inspection of the pictures. In adult participants, impoverished control of selective attention led to lower sampling of informative parts of the stimulus (Tsal & Kolbet, 1985) and could explain variance in the picture morphing task (Ruggieri & Fernandez, 1994). Also, fixations recorded prior to object recognition predicted which interpretation of an ambiguous image was reported (Kietzmann, Geuter, & König, 2011). Wimmer and Doherty (2007), however, failed to find any difference in eye movement patterns between children who reversed an ambiguous figure and children who failed to do so.

Results of Experiment 1 revealed that children in all age groups required more morphs to switch interpretations than adult controls, with no significant improvements within the child group. In Experiment 2, we tested older children (up to 9 years) to examine at which age the ability to report the second object in the picture morphing task improves.

## Experiment 1

### Participants

#### Children

Participants were 66 3- to 5-year olds (*M*_age_ = 54.95 months; SD = 10.46, age range 37–71, 33 girls) from five nurseries in Austria and Germany. Parents gave written consent and the children gave their assent to participation. Seventeen additional children failed to complete at least two sets of the picture morphing task (see “Procedure and materials”) and were excluded.

#### Adults

Seventy-six participants (*M*_age_ = 36.82 years, SD = 9.07; age range 22–61 years; 30 females) were recruited through Mechanical Turk. All participants gave informed written consent prior to participation by clicking on the “I agree” button. Five additional participants were excluded because they quit the task prematurely within the first set (*n* = 2) or failed to complete at least two sets of the picture morphing task (*n* = 3). Participants received $2 for their participation. Ethical approval was granted by the Ethics Panel of the University of Salzburg, following the principles expressed in the Declaration of Helsinki.

### Design

**Fig. 1:**
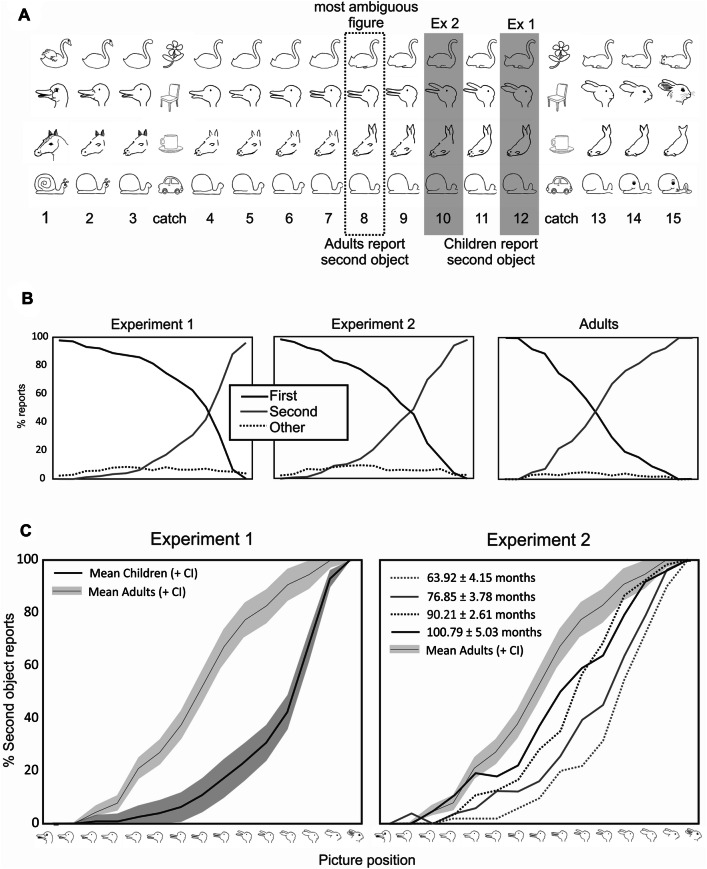
**a** Stimuli in the picture morphing task. **b** Overall percentages of reports for children in Experiment 1 (left), children in Experiment 2 (center) and adult participants (right)—averaged over all picture sets. The *x*-axis represents the gradual morph from the first object (100% the first object) to the second object (0% first object). The black solid line represents the responses (in %) identifying the first object, the gray solid line displays the responses identifying the second object. The dashed line represents answers other than the first or second object. **c** Average percentage of participants reporting the second object (*y*-axis) at each of the fifteen morphing stages (*x*-axis) for valid picture sets for Experiment 1 (left) and Experiment 2 (right)

Each participant was exposed to four sets of the picture morphing task (Fig. 1a). Children additionally received (a) a brief check that they knew the names of the animals in the task prior to the morphing task, (b) an unexpected transfer false belief task (Perner, Mauer, & Hildenbrand, 2011) and (c) a day/night Stroop task (Gerstadt et al., 1994). Half of the children received the picture morphing task prior to the false belief task and the day/night Stroop task; half had the reverse order. The order of the false belief task and the Stroop task was fully counterbalanced. Testing of children lasted up to 30 min and took place in a quiet room at children’s nurseries. Adult controls needed on average 11 ± 6 min to complete the task.

### Procedure and materials

#### Picture morphing task

Four sets of line drawings were used (Fig. 1a), two (duck/rabbit and swan/cat) were taken from Stöttinger et al. (2014), and two (horse/seal and snail/whale) were created by the last author in Paint©. Each set was based on a well-known ambiguous figure (Bernstein & Cooper, 1997; Fisher, 1968; Jastrow, 1900; Wittgenstein, 1953). Over 15 iterations, an unambiguous representation of an animal (e.g., a duck) morphed into an unambiguous representation of a different animal (e.g., rabbit). The eighth picture of each set represented the most ambiguous figure. At the fourth and fourteenth positions, a ‘catch’ object was included to evaluate whether participants simply perseverated on a single response. Pictures (for children: 12 × 12 cm; for adults: 300 × 300 pixels) were presented on a computer monitor one at a time (black on white background). Set order followed a Latin Square Design. Morphing direction (e.g., duck to rabbit vs. rabbit to duck) was fully counterbalanced.

Participants were told that they would see the picture of an animal gradually changing into another animal. They were asked to verbally state what they saw (children) or to type in the word (adults) for each picture. Answers were rated as first or second object when they fit the general concept (e.g., swan, duck, bird, stork, etc. were rated as “swan”). Answers not matching the concept (e.g., “snake”), and omissions, were categorized as “other”. The categorization was done independently by two raters with an interrater agreement of 98.1%. Cases of disagreement were discussed and resolved to mutual satisfaction. The dependent variable was the picture position at which participants reported the second object.

Sets were coded as completed, following three criteria: (1) both catch trials were identified correctly; (2) the second interpretation was identified correctly at least once; and (3) no “other” reports were made.

In children, eye movements were recorded throughout the picture morphing task to test whether second object interpretations were related to their visual inspection of the drawings. Eye movements were recorded monocularly from the right eye with an EyeLink 1000 (SR-Research, Ontario, Canada) eye tracker at a sampling rate of 500 Hz. We used the “remote” setup that did not require head stabilization but tracked a target sticker on the children’s forehead. The viewing distance was approximately 50 cm. Stimuli were presented on a 17-in. CRT-monitor with a resolution of 1024 × 768 pixel and a frame rate of 60 Hz. A three-point calibration routine preceded the experiment. Each picture set was preceded by a fixation control for which the children had to fixate a centrally presented cross for a minimum duration of 150 ms. If the fixation control procedure failed, the system was re-calibrated. Thereafter, the picture morphing task was presented one picture at a time. Each picture was presented until the child gave a response. Eye-tracking data were analyzed with R (R Core Team, 2019). We considered mean number of fixations, mean fixation duration (excluding fixations of less than 80 ms) and mean summed saccade length per picture in each sequence as dependent measures.

#### Unexpected transfer false belief task

We used a PowerPoint© version of the unexpected transfer false belief task (Perner et al., 2011). A female doll, Lisa, puts a teddy into a red box and leaves. A male doll, Tom, enters, transfers the teddy into a yellow box and leaves. Children were asked four Comprehension Questions: (1) where did Lisa place the teddy? (2) Where is the teddy now? (3) Who placed it there? and (4) did Lisa see that? If children gave one or more incorrect answers, the entire scenario was repeated until all control questions were answered correctly (*n *= 18). Finally, Lisa returned and children were asked where she will look first for her teddy (prediction question).

Regardless of whether children made a correct prediction or not, Lisa was then shown to search in the red box and children were asked to explain her behavior (explanation question). Following Wimmer and Mayringer (1998), explanations were considered correct when they either contained appropriate mental state words (e.g., “she *thought* the teddy was in there”; “she did not *see* it being moved”) or relevant story facts (e.g., “she had put it in the red box”; “somebody has taken the teddy away”). All other answers were categorized as incorrect.

#### Day/night Stroop task

We used one set of cards (13.5 × 10 cm) following the day/night Stroop task by Gerstadt et al., (1994). Children were instructed to say “day” when shown a black card with a white moon and stars and to say “night” when shown a white card with a yellow sun. Subsequently, children were presented with a total of 16 cards in the order night (n), day (d), d, n, d, n, n, d, d, n, d, n, n, d, n, d. If children responded incorrectly on one of the first two trials, they were reminded of the rules (two times at maximum) and the test was restarted. Thereafter, children received no direct feedback. The dependent measure was the number of trials answered correctly.

## Results

### Picture morphing task

Only complete picture sets were included in the analysis (see Procedure and Materials section for more details). Three percent of all responses in adults and 6% of all responses in children comprised “other” responses, equally distributed over the course of the task (Fig. 1b).

The most excluded picture set was the cat/swan set, but exclusion rates were comparable between children and adults for each picture set (cat/swan: 32% children, 26% adults; duck/rabbit: 8% children, 7% adults; horse/seal: 14% children, 9% adults; snail/whale: 18% children, 17% adults). On average, children completed 3.29 ± 0.72 sets (2 sets: *n* = 10; 3 sets: *n* = 27; 4 sets:  *n* = 29), adults 3.41 ± 0.75 sets (2 sets:  *n* = 12; 3 sets: *n* = 21; 4 sets: *n* = 43), with both participant groups being worse at reversing the horse/seal picture set compared to the other three sets.^1^ As participants progressed from set 1 through to set 4, the average number at which participants identified the second object stayed relatively constant,^2^ meaning that participants did not improve throughout the task (Figure 1 in Supplemental Materials).

Finally, the average picture number at which a switch was reported across all valid sets was submitted to an analysis of variance (ANOVA) for participant group (children vs. adults). This analysis revealed a highly significant main effect [*F*(1, 140) = 137.23, *p* < 0.001, *η*^2^= 0.50]. Adults identified the second object on average at picture #8 (*M* = 8.38, SD = 1.89), and therefore significantly earlier than children, who identified the second object at around picture #12 (*M* = 11.99, SD = 1.76) (Fig. 1a, c, left panel).

### Correlation between performance in the picture morphing task and other cognitive abilities

In the false belief test, 45 (68%) children correctly predicted that Lisa would search in the empty location (prediction question). Regardless of whether children made a correct prediction or not, they were asked to explain her behavior (explanation question). Twenty-eight children (42%) correctly answered the explanation question, the majority of whom (*n* = 23) referred to relevant story facts. In the day/night Stroop inhibitory control task, out of 16 possible responses, children gave 10.8 (SD = 5.01) correct answers on average. To test whether there is a connection between performance in the false belief task (correct prediction and explanation), day/night Stroop task and picture morphing task (average number of first objects reports for valid sets), we calculated correlations as well as partial correlations controlling for age (Table 1).

**Table 1:** Pairwise correlations [partial correlations controlling for age] between picture morphing task, false belief task (separate for prediction and explanation question), day/night Stroop task, and age

	1	2	3	4
1. Picture morphing	–	[0.069]	[− 0.094]	[0.009]
2. Prediction	0.001	–	[0.081]	[− 0.044]
3. Explanation	− 0.146	0.257*	–	[0.018]
4. Day/night Stroop	− 0.051	0.143	0.200	–
5. Age (months)	− 0.141	0.430***	0.447***	0.416**

^+^*p *<0.1, *p <0.05, **p <0.01, ***p ≤ 0.001 (two-tailed)

Age correlated with all measures except performance in the picture morphing task. Performance in the prediction and explanation question also correlated moderately, but this fell short of significance when age was controlled for (upper half of Table 1). No other correlations were significant.

### Eye-tracking data

**Fig. 2:**
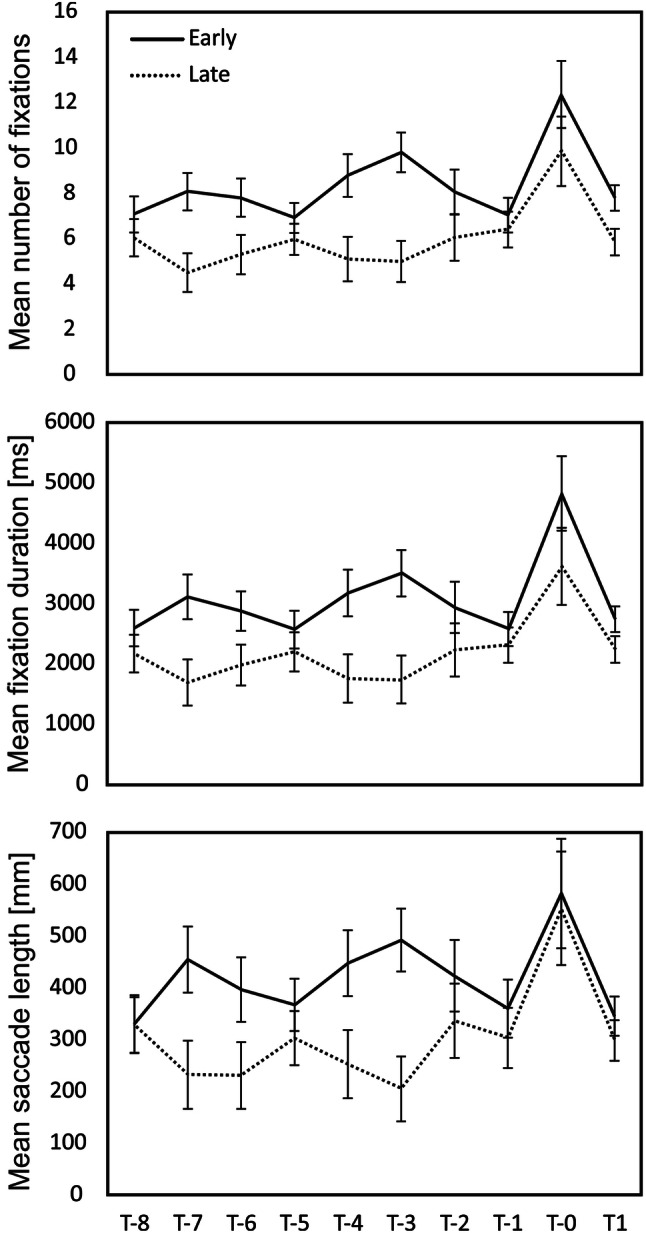
Mean number of fixations, mean fixation duration (in ms), and summed saccade length (in mm) for eight pictures prior to the switch (T-8 to T-1), at the switch (T0), and one picture after the switch (T1), separate for “early” (≤ 11 pictures) and “late” switchers (> 11 pictures) in the picture morphing task. Error bars represent the standard error of the mean (SEM)

A median split was used to divide children into “early” (i.e., ≤ 11 pictures; *M* = 9.72 ± 1.50) and “late” switchers (i.e., > 11 pictures; *M* = 12.33 ± 0.66 pictures) based on the average number of first object reports they made before reporting the second object in the picture morphing task. Figure 2 shows mean number of fixations, mean fixation duration, and mean summed saccade length for eight pictures prior to the switch (T-8 to T-1), at the switch (T0), and one picture after the switch (T1).

Data were submitted to three separate mixed factorial repeated measures analyses of variance (ANOVA)^3^ for (1) mean number of fixations, (2) mean fixation duration, and (3) mean summed saccade length as dependent variables and time point of switch (i.e., the eight pictures prior to the switch, the picture at the switch as well as one picture after the switch) as the within-subjects factor and performance group (early vs. late) as the between-subjects factor. Analyses revealed significant main effects for time of switch and performance group: the average number of fixations [*F*(9, 504) = 6.36, *p* < 0.001, *η*^2^= 0.10], the average fixation duration [*F*(9, 504) = 6.15, *p* < 0.001, *η*^2^= 0.10], as well as the saccade length [*F*(9, 504) = 3.64, *p* < 0.001, *η*^2^= 0.06], all peaked at the moment of the switch (pairwise—Bonferroni corrected—*t* test for post hoc analyses; highest *p* value = 0.027; Fig. 2). Early switchers showed significantly larger number of fixations [*F*(1, 56) = 10.05, *p* < 0.01, *η*^2^= 0.15], longer mean fixation durations [*F*(1, 56) = 7.31, *p* < 0.01, *η*^2^= 0.12], and longer saccades [*F*(1, 56) = 4.24, *p* < 0.05, *η*^2^= 0.07]. None of the interactions between time × performance group reached significance (all *p*s > 0.15), indicating that the two groups did not differ in their inspection patterns over time.

**Fig. 3:**
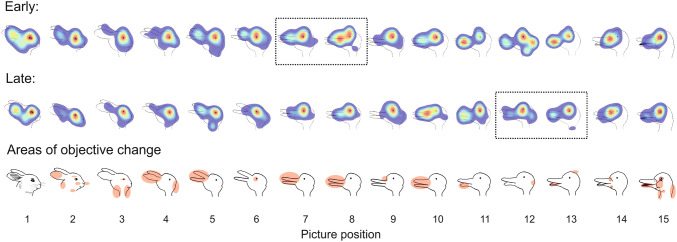
Heat maps of the cumulative number of fixations displayed separately for “early” (≤ 11 pictures, top) and “late” switchers (> 11 pictures, center) with warmer colors indicating more attention. (For interpretation of the references to color, the reader is referred to the web version of this article.). The dotted squares represent where participants on average reported a shift in perception. The bottom row highlights the area of change from one picture to the next

For exploratory analysis, we created heat maps for the rabbit/duck picture-set. This set was selected because it had the highest number of valid data sets and the highest number of eye tracking data sets available (*n* = 29). Also, there was considerable variance between participants which allowed us to divide the sample into early (*n *= 13) and late switching groups (*n* = 16) based on where children reported the second object in this particular set. Children in the early switching group reported seeing the duck between picture #7 and #8 (*M* = 7.54, SD = 1.76). This was on average one to two pictures later compared to adults’ performance in the same picture-set [*M *= 5.82, SD = 1.52; *t*(50) = 3.39, *p* < 0.01]. The late switching group reported seeing the duck on average between picture #12 and #13 (*M* = 12.56, SD = 1.32), therefore significantly later than adults [*t*(53) = 15.51, *p* < 0.001] and early switching children [*t*(27) = 8.80, *p* < 0.001]. Heat maps were created for each of the fifteen pictures—separately displayed for the early and late switching group (Fig. 3). These heat maps color-code the density of fixation locations (weighted with regards to the duration of the fixation). In other words, they illustrate the allocation of visual attention to the pictures with warmer colors indicating more attention. As evident in Fig. 3, children who recognized the duck later focused mainly on the eye for the first nine pictures; whereas, children who identified the duck earlier allocated their attention also on a second area (i.e., the area around the beak). Also, earlier switching children seemed to be more attentive to actual changes between the pictures (e.g., picture #11, #12, and #13) than later switching children—whose attention was again mostly drawn towards the eye of the figure.

## Discussion

Children overall reported the second object much later (i.e., at picture #12) than adults (i.e., at picture #8) and only when the picture displayed predominantly the second object (Fig. 1a). Also, there was no clear developmental improvement despite older children outperforming younger children on theory of mind and inhibitory control (Table 1). While these factors have affected the probability of content informed reversal in previous studies (Bialystok & Shapero, 2005; Doherty & Wimmer, 2005; Gopnik & Rosati, 2001; Mitroff et al., 2006; but see Ropar Mitchell, & Ackroyd, 2003), none of these measures affected performance on the picture morphing task (i.e., when children were naïve as to the second interpretation, but were continuously shifted towards that interpretation).

It is plausible to assume that children switched so late due to the rather impoverished silhouette type displays of objects. While we cannot fully rule out that children struggled more than adults with the stimuli, all children were able to name animals based on line drawings in the word naming check prior to the picture morphing task. Although stimuli in the middle were highly ambiguous and suggestive of more than one interpretation, “other” reports for children (and adults) did not peak at the most ambiguous figure. Instead, they were equally distributed across all picture positions (Fig. 1b). Critically, adults gave similar “other” reports as did children (e.g., “dinosaur” in the cat–swan picture set) and a comparable proportion of sets had to be excluded in both participant groups. Also, children and adults found it harder to reverse the horse–seal picture set compared to the other sets.

One may argue that the word naming check prior to the picture morphing task primed children to search for those same animals. This, however, should have made the task easier for children and cannot explain their delayed recognition of the second object. Children who used more extensive search strategies—as suggested by longer and a higher number of fixations as well as longer saccades—identified the second object earlier. For one of the picture sets, qualitative analysis of fixation patterns revealed an interesting trend. Children who switched later focused mainly on the eye of the figure, while children who recognized the second object earlier were also able to direct their attention to other regions. They still, however, did not reach adult level performance. Children in all age groups required more pictures before they reported the second object compared to adult participants, with no significant age improvements.

In Experiment 2, we, therefore, extended the age (up to 9 years) to examine at which age the ability to report the second object in the picture morphing task improves. In addition, we measured theory of mind and inhibitory skills, using a different type of measurement. Advanced theory of mind skills were measured using Happé’s strange stories (O’Hare, Bremner, Nash, Happé, & Pettigrew, 2009). This task was previously used to assess mentalizing skills in middle childhood (Devine & Hughes, 2013) and was consequently regarded as an appropriate task for our sample. Further, because the day/night Stroop task by Gerstadt et al., (1994) was not related to the picture morphing task, we substituted the Stroop task with the Simon task (picture test) by Davidson, Amso, Anderson, and Diamond (2006). This task is also appropriate for the age range tested but assesses slightly different skills. While the Stroop task in Experiment 1 requires the inhibition of conceptually incompatible stimuli, which is expected to be developed in middle childhood, participants in the Simon task need to inhibit responses that are spatially incompatible with the stimuli. In other words, it examines competition at the stimulus response level (Liu, Banich, Jacobson, & Tanabe, 2004), which we deem relevant in our task where local changes increasingly fail to match the global representation of the animal.

Although qualitative analysis of fixation patterns revealed an interesting trend, we did not include eye-tracking metrics in the follow-up experiment. We were able to confidently measure number and duration of fixations as well as saccade length in Experiment 1. Yet, the restricted number of valid sets for which we had eye tracking data only allowed us to link fixation location with the actual interpretation of the picture in one picture set in Experiment 1. While we would have had enough data for a similar analysis in two other picture sets (i.e., snail–whale: *n* = 27; horse–seal: *n* = 29), these sets did not produce enough variance in responses to separate groups into early and late switchers. That is, more than 80% of participants reported the second object at—or later than—picture #11. For meaningful insights from an ROI analysis we would have to select new picture sets that produce more variance. Because we further wanted to establish at which age children would reach adult level performance, we opted against changing the picture sets in Experiment 2 that would have allowed for such analysis.

## Experiment 2

### Participants

Fifty-four 4- to 9-year olds (*M*_age_ = 83.41 months; SD = 14.52, age range 55–113, 26 girls) from five nurseries and one school in Germany took part. Three additional children failed to complete at least two sets of the picture morphing task and were excluded. Children’s parents gave written informed consent. Ethical approval was granted by the Ethics Panel of the University of Salzburg, following the principles expressed in the Declaration of Helsinki.

For later analysis, we spilt the sample into four similar-sized groups: 5.5-year olds: (*n* = 13, *M*_age_ = 63.79 months, age range 55–70), 6.5-year olds (*n* = 13, *M*_age_ = 76.85 months, age range 72–85), 7.5-year olds (*n* = 14, *M*_age_ = 90.21 months, age range 86–93), and 8.5-year olds (*n* = 14, *M*_age_ = 100.79 months, age range 94–113).

### Design

Each child was exposed to the picture morphing task of Experiment 1. A test of advanced theory of mind (strange stories suitable for 4- to 9-year olds, Happé, 1994; O’Hare, Bremner, Nash, Happé, & Pettigrew, 2009) and a Simon task (picture test, Davidson et al., 2006) were administered. Task order followed a Latin square design.

### Procedure and materials

#### Test of advanced theory of mind

Three strange stories based on Happé (1994) were administered: lie (dentist), forget (doll) and misunderstanding (glove). We selected these three stories because they showed variance in performance even in 5-year olds (O’Hare et al., 2009). For example, in the dentist story children were told about John, who hates going to the dentist. Anytime John has toothache, he needs a filling and that hurts. At the moment John has bad toothache but when his mother asks him whether he has toothache he says “No, Mummy.” Protagonists in these stories made statements they did not mean literally, and children were asked “Was it true what X said?” and “Why did X say this?” Scoring followed O’Hare et al. (2009).

#### Simon task

Color pictures of frogs and butterflies appeared on the left or right side of the computer screen. Children were instructed to press the green bell, if they saw a frog, and the pink bell, if they saw a butterfly. In congruent trials, animals appeared on the same side as the assigned bell; in incongruent trials, they appeared on the opposite side, and in mixed trials on both sides. Every condition consisted of 20 pictures, presented for 750 ms each, followed by a black fixation cross. Children received an initial practice with five pictures. We used accuracy (i.e., pressing the green bell upon a frog’s appearance, and the pink bell upon the butterfly’s appearance) as the measure.

## Results

### Picture morphing

Seven percent of all responses comprised “other” responses, equally distributed over the course of the task (Fig. 1b, middle panel). The most sets had to be excluded in the cat/swan (22%) and snail/whale (22%) picture sets with only a few exclusions for duck/rabbit (4%) and horse/seal (7%). On average, children completed 3.44 ± 0.74 sets (2 sets: *n* = 8; 3 sets: *n* = 14; 4 sets: *n* = 32). As they progressed from set 1 through to set 4, the average number at which they identified the second object stayed relatively constant.^4^ As in Experiment 1, children recognized the second object later in the horse–seal picture set compared to the other three sets.^5^

As in Experiment 1, the picture number at which children reported the second object was averaged across all valid sets and compared against performance of adults in Experiment 1. This analysis of variance (ANOVA) for participant group (children vs. adults) revealed a highly significant difference between children and adults [*F*(1, 128) = 40.77, *p* = 0.001, *η*^2^= 0.24]. Children reported the second object on average two pictures later (*M* = 10.54, SD = 1.91) than adults (*M* = 8.38, SD = 1.89) (Fig. 1a).

Restricting the ANOVA to children (5.5-year olds, 6.5-year olds, 7.5-year olds, and 8.5-year olds with 13–14 children in each group) showed that the youngest children (*M* = 11.89, SD = 1.06) needed significantly more pictures to identify the emerging animal compared to the 7.5-year olds (*M* = 9.92, SD = 1.79) and the oldest children (*M* = 9.49, SD= 2.07) [*F*(3, 50) = 5.36, *p* = 0.003, *η*^2^= 0.24; post hoc Bonferroni corrected all *p*’s< 0.05]. No significant difference was found between 5.5- and 6.5-year olds (*M* = 10.98, SD = 1.72; post hoc Bonferroni: *p* = 1) (Fig. 1c, right panel). The first three age groups reported the second object significantly later than adult participants (post hoc Bonferroni corrected all *p*’s < 0.05), with the oldest age group approaching adult level performance (post hoc Bonferroni corrected *p* = 0.20).

### Correlation between performance in the picture morphing task with other cognitive abilities

Children achieved a mean score of 4.35 (SD = 1.78) out of a possible maximum score of 6 in the test of advanced theory of mind. In the Simon task, children made significantly fewer errors in congruent trials (*M* = 1.46, SD= 2.44) than in incongruent trials (*M* = 2.54, SD= 2.87), *t*(51) = 3.87, *p* < 0.001, or mixed trials (*M* = 2.44, SD= 3.27), *t*(51) = 3.41, *p* = 0.001, which did not differ, *t*(51) = 0.26, *p* = 0.79. We again calculated correlations and partial correlations (controlling for age) to assess a connection between inhibitory control and theory of mind with performance in the picture morphing task.

Age correlated with all measures. Performance in the picture morphing task correlated with all measures in the Simon task. Theory of mind also correlated with performance in the congruent and incongruent trials of the Simon task. None of these correlations remained significant after controlling for age (upper half of Table 2).

**Table 2:** Correlations [partial correlations controlling for age] between picture morphing, theory of mind, Simon task (congruent, incongruent and mixed), and age in Experiment 2

	1	2	3	3a	3b	3c
1. Picture morphing	–	[− 0.015]	[0.111]	[0.115]	[0.080]	[0.098]
2. Theory of mind	− 0.208	–	[− 0.132]	[− 0.138]	[− 0.097]	[− 0.113]
3. Simon task	0.349*	− 0.321*	–	[0.880***]	[0.824***]	[0.889***]
(a) Congruent	0.364**	− 0.336*	0.917***	–	[0.621***]	[0.719***]
(b) Incongruent	0.301*	− 0.275*	0.870***	0.726***	–	[0.532***]
(c) Mixed	0.285*	− 0.265^+^	0.906***	0.777***	0.628***	–
4. Age (months)	− 0.516***	0.402**	− 0.547***	− 0.579***	− 0.491***	− 0.426***

^+^*p *<0.1, *p <0.05, **p <0.01, ***p ≤ 0.001 (two-tailed)

## Discussion

The aim of the second experiment was to look at the developmental trajectory in the picture morphing task beyond pre-school age and to further investigate the relation with theory of mind and inhibitory skills. The ability to identify the second object improved reliably from 4 years to 9 years, with older children needing significantly fewer pictures to update their representation of the visual stimuli compared to younger children. Replicating Experiment 1, we again failed to find a connection with inhibition and theory of mind despite using a refined measurement. Also, children were able to identify catch trials successfully, showing that they are in principle capable of inhibiting prepotent responses when faced with a completely different image. It is, therefore, unlikely that inhibitory deficits can account for the developmental effects found in our study.

## General discussion

To date, there are only a handful studies investigating how children reverse ambiguous figures. These studies typically find that young children never spontaneously reverse (Girgus et al., 1977; Gopnik & Rosati, 2001; Mitroff et al., 2006; Rock & Mitchener, 1992; Wimmer & Doherty, 2011) and even 10-year olds still show lower reversal rates than adults (Ehlers et al., 2016). Children until the age of five are only able to alternate between two interpretations of an ambiguous figure when they are informed about the second interpretation beforehand (i.e., content informed reversal). The present set of studies used a continuous version of the ambiguous figures task to measure how children switch to a second interpretation under conditions where they were naïve as to the second object and where each change in the picture increasingly supported the alternative interpretation.

In both experiments, we found that children switched much later than adult participants. Performance in the picture morphing task improved with age—as demonstrated in Experiment 2—with 8.5-year olds (*M* = 9.49) starting to approach the level of adult participants (*M* = 8.38). Our results are, therefore, in line with earlier findings showing an improvement with age on reversals of ambiguous figures (Ehlers et al., 2016; Girgus et al., 1977; Gopnik & Rosati, 2001; Mitroff et al., 2006; Rock & Mitchener, 1992; Wimmer & Doherty, 2011).

We further investigated whether children who possessed advanced theory of mind understanding and strong inhibitory skills would recognize the second object sooner in the picture morphing task. None of these measures predicted performance in our continuous ambiguous figures task. Previous research has found inconsistent evidence for a link between reversals and false belief, with two studies showing a link (Gopnik & Rosati, 2001; Mitroff et al., 2006); while more recent evidence (Doherty & Wimmer, 2005; Wimmer & Doherty, 2011) failed to find a direct link between false belief and reversals. Studies that found a relationship interpreted the link as reflecting a common reliance on actively contrasting perspectives. The late switch to the second object in the picture morphing series, however, suggests that switching requires a strong external drive in young children (i.e., when the picture represents predominantly the second object) which may not rely on the ability to abstractly contrast perspectives.

Instead, stronger inhibitory skills facilitated reversals in a previous set of studies (Wimmer & Doherty, 2011) which suggested that only if the current interpretation can be inhibited, can the second interpretation be identified. Critically, performance can either benefit from a top–down inhibitory insight (i.e., knowing what to inhibit) or from a more general bottom–up inhibitory strength (i.e., having enough power to inhibit; Wimmer & Doherty, 2011). If performance was predicted by the Simon task (Experiment 2), but not by the Stroop task (Experiment 1), it would favor the latter explanation. While the Stroop task assesses the ability to inhibit cognitive interference, the Simon task requires interference resolution at the stimulus response level. Neither the correlation with the Stroop task (Experiment 1) nor the correlation with the Simon task (Experiment 2) remained significant after controlling for age, which suggests that improvements in the picture morphing task are not explained by improvements in inhibitory strength.

The late shift in our child sample may instead reflect limitations in working memory capacity, particularly as working memory measures are typically correlated with inhibition measures (Davidson et al., 2006). Indeed, switching to a new interpretation requires mental manipulation. In addition to perceiving the picture, participants have to go through different interpretation alternatives at the same time, a process that particularly young children may find too taxing. Data from a pilot study, however, speak against this explanation. There we presented 5- to 6-year-old children^6^ with a manual version of this task where we asked them to sort the pictures into a “rabbit” or a “duck” box. Despite a clear reduction in working memory load (i.e., children had to simply compare the picture with the target pictures on the boxes), children still needed significantly more pictures before they reported the second object compared to adult participants reported in Stöttinger, Guay, Danckert and Anderson (2018); [*F*(1, 115) = 21.50, *p* < 0.001, *η*^2^ = 0.16]).

The exceptionally late shift could potentially reflect a motivation issue, especially in our youngest children. Odic, Hock, and Halberda (2014) showed that a history of difficult, low-confidence perceptual decisions resulted in degraded performance in subsequent easier decisions in 4- to 5-year-old children. This perceptual hysteresis (i.e., “…the persistence of the initially established percept despite the evidence reaching values that favor the alternative percept”; p. 2; Odic et al., 2014) was explained by a tendency to guess without paying attention to the actual sensory input. Only when the task was easy enough did they re-attend. Although a lack of trying could potentially account for our data, Odic et al. (2014) only found evidence for perceptual hysteresis when children were provided with explicit (i.e., correct/incorrect) feedback. This effect disappeared after explicit feedback was removed. Given that no explicit feedback was provided in our picture morphing task, we deem it unlikely that low motivation due to constant discouraging feedback could fully account for our data. Also, our eye tracking data confirm that children were paying attention to the stimuli throughout the procedure.

It is also possible that the delayed switch to the second interpretation in the picture morphing task reflects a deeper conceptual issue in children. French, Menendez, Herrmann, Evans, and Rosengren (2018), for example, showed that children (in particular) and adults (to a lesser extent) had persistent problems reasoning about certain types of changes in biological organism that occur over the life-span (e.g., the metamorphosis of a caterpillar changing into a butterfly). Confronted with an unfamiliar animal, children and adults apply the default cognitive constraint that physical features remain stable across the life-cycle. This constraint helps us to maintain an image of a stable world in which ducks do not change into rabbits. The acknowledgement of such metamorphosis increases with age as children become more familiar with the concept. This could explain why younger children retained a stronger bias to resist such changes than older children and adults.

Alternatively, eye tracking data of Experiment 1 showed that children who employed a more extensive exploration strategy (i.e., longer and higher number of fixations in Experiment 1) identified the second object earlier. Typically, a larger number of fixations are associated with being a novice to a scene (Kelly, Rainford, Darcy, Kavanagh, & Toomey, 2016), thus requiring more fixations to cover the visual space. In this respect, it is plausible that more fixations indicate acknowledging novelty to a stimulus in our task. However, none of our children were experts, and it is, therefore, possible that a larger number of fixations have led to a benefit of finding crucial cues. A focus on specific parts of an ambiguous stimulus (e.g., the rabbit’s ears) will favor one interpretation (rabbit) over the other (Tsal & Kolbet, 1985). Although Wimmer and Doherty (2007) failed to find evidence for a causal relation between search patterns and reversals of ambiguous figures in 3- to 5-year olds, our qualitative ROI analysis did show an interesting trend: children who recognized the duck earlier showed a broader allocation of attention than children who identified the duck later. It remains unclear why some children adopted a more extensive looking pattern than others. None of the indicators that we had in mind (i.e., false belief and inhibition) correlated with eye-tracking measures.

However, even if children benefited from a higher number of fixations, and a more diverse looking pattern, only the oldes children started to approach adult level performance. Assuming that scanning strategies were not the main causal factor for this difference, successful recognition of the emerging animal may additionally require high-level (cognitive) search strategies. Adult participants consistently report the target object at a point when the picture still represents more the first object than the second. Interestingly, this is only the case when the picture is presented in a gradual context compared to when the same picture is presented outside of the morphing context (Egré, Ripley, & Verheyen, 2018; Stöttinger et al. 2018; Stöttinger, Sepahvand, Danckert, & Anderson, 2016). This effect is reversed after damage to the right hemisphere: right-brain-damaged patients need significantly more pictures to identify the emerging object when it is presented in the gradual compared to the individual condition (Stöttinger et al., 2018). It is speculated that healthy adult participants use high-level “exploratory” search strategies (e.g., it started out as a duck, but what else could it be?)—an exploration strategy that may be impaired after damage to the right hemisphere (Danckert et al., 2012; Mohammadi Sepahvand, Stöttinger, Danckert, & Anderson, 2014). Such exploration strategies may also be late developing in children.

The very late shift in children and in right-brain-damaged patients might be the result of a fundamentally different exploration strategy compared to healthy adults. When one object gradually morphs into another in the picture morphing task, focusing on locally changing elements does not support identification of the object. Only the ability to impose a holistic, “Gestalt-like” template (Van de Cruys et al., 2014) of an imagined object onto the current item enables the exploration of potential fits (see Rock et al. 1994; Wimmer & Doherty, 2011 for the same argument in the context of ambiguous figures). A local bias can explain why children and right-brain-damaged patients struggle to recognize the second object. The suggestion here is that for the patients, damage to the right hemisphere has disrupted global processing; while for the children, these processes have yet to fully develop (Moses et al., 2002 for a review). For example, when confronted with a shape (e.g., the letter H) composed of smaller elements (e.g., small As), right-brain-damaged patients are able to remember details but not the overall pattern, while results are reversed in patients with a damage to the left side of the brain (Delis, Robertson, & Efron, 1986). Similarly, right-brain-damaged patients typically comment on the local changes in the picture morphing task (e.g., duck, duck with beak open, duck with a wider beak, duck looking up, etc.) but struggle to shift to a new interpretation (i.e., rabbit; Stöttinger et al., 2014). The same local over global bias is found in children. For example, 6-year olds use a local “piecemeal approach” when asked to copy a Rey–Osterrieth Complex Figure. That is, they tend to copy one small detail after the other instead of starting with the global shape and adding details afterwards (Akshoomoff & Stiles, 1995a; Akshoomoff & Stiles, 1995b; Martens, Hurks, & Jolles, 2014). In a similar vein, when confronted with the Kanizsa illusion (i.e., black “pacman” shapes arranged to induce a contour illusion of a bright white triangle in the middle) 4-year olds focus more on the local—illusion inducing “pacman” shapes, while 7-year old children show an adult-like bias towards the global “illusory middle shape” (Nayar, Franchak, Adolph, & Kiorpes, 2015). This shift from a local to a global processing style is accompanied by a developing hemispheric specialization in the brain. Moses et al. (2002), for example, showed that children who performed like adults in a hierarchical figure task (i.e., global shapes composed of smaller elements) also showed the same adult-like right hemispheric preference for global processing. While some studies report a transition from local to global processing around 6 years of age (Dukette & Stiles, 1996; Kimchi, Hadad, Behrmann, & Palmer, 2005; Martens et al., 2014; Poirel, Mellet, Houdé, & Pineau, 2008), others find evidence for development further into the teenage years (see Nayar et al., 2015 for a review). Qualitative ROI pattern analysis of our eye-tracking data supports the idea that a local bias protracts identification of the second object in the picture morphing task. Children who struggled to identify the duck in the “duck–rabbit” picture set did indeed focus mainly on one detail (i.e., eye; Fig. 3).

In two separate studies, we showed that children gradually improve beyond pre-school age in a continuous version of the ambiguous figures task—a finding that was highly reliable. We speculate that a local over global exploration style can account for our findings. Future studies will have to investigate whether it is indeed a local processing style that hinders children to switch to the second interpretation, or whether children’s ability to explore and generate plausible alternative interpretations or the default heuristic of assuming feature stability is causing the delay in their performance.

## Electronic supplementary material

Below is the link to the electronic supplementary material.

Supplementary material 1 (DOCX 94 kb)

## Data Availability

The data that support the findings of this study are available on OSF “Continuous ambiguous figures task” at https://osf.io/jx5nt/.

## Footnotes

1 A mixed-effect ANOVA for the average picture number at which a switch was reported was calculated with the picture set (duck–rabbit, cat–swan, horse–seal, snail–whale) as a within-subjects factor and participant group (children vs. adults) as a between-subjects factor. This analysis revealed a significant main effect for participant group [*F*(1, 70) = 83.68, *p* < 0.001; *η*^2^ = 0.54], and picture set [*F*(3, 210) = 6.47, *p* < 0.001; *η*^2^ = 0.09], but no significant interaction between participant group × picture set [*F*(3, 210) = 0.49, *p* > 0.65, *µ*^2^ = 0.007]. Overall, participants identified the second object later in the horse/seal picture set compared to all other sets (pairwise—Bonferroni corrected—*t* test; smallest *p* value = 0.012). Note, this analysis comprises a smaller sample, because only 29 children and 43 adults completed all four sets.

2 The same mixed-effect ANOVA was calculated for set order (set 1–set 4) as the within-subjects factor. This analysis revealed a significant main effect for participant group only [*F*(1, 70) = 83.68, *p* < 0.001; *η*^2^ = 0.54] with neither a significant effect for set order [*F*(3, 210) = 1.28, *p* > 0.25; *η*^2^ = 0.02], nor a significant interaction between set order × participant group [*F*(3, 210) = 1.16, *p* > 0.30; *η*^2^ = 0.02].

3 Note that data were not available for fourteen children due to problems during the calibration procedure. Behavioral data for the remaining 52 children (*M*_age_ = 55.92 months) closely resembles results for the entire group (i.e., Median = 11; Mean_high performers_ = 9.86 ± 1.20; Mean_low performers_ = 12.22 ± 0.65).

4 A repeated-measures analysis for the average picture number at which a switch was reported was calculated for set order (set 1–set 4) as independent variable. This analysis revealed a small but significant effect [*F*(3, 93) = 2.76, *p* = 0.046; *η*^2^ = 0.08] with a benefit for set 3 compared to the other sets. This effect, however, did not survive Bonferroni correction for multiple comparison.

5 A repeated-measures analysis for picture set (duck–rabbit, cat–swan, horse–seal, snail–whale) showed a significant effect: *F*(3, 93) = 5.92, *p* < 0.01; *η*^2^ = 0.16. Children identified the second object later in the horse–seal picture set compared to all other picture sets (pairwise *t* test; all *p*’s< 0.05), although the comparison of the horse–seal vs. snail–whale sets fell short of significance after Bonferroni correction.

6 *N* = 40; *M*_age_ = 66.90 ± 4.25 months; 19 girls.
