# Supplementary material for: Children struggle beyond preschool-age in a continuous version of the ambiguous figures task
Source: Psychol Res. 2019 Dec 19;85(2):828–41. doi: 10.1007/s00426-019-01278-z (PMC7900074; doi:10.1007/s00426-019-01278-z)
Supplement: Supplementary file 1 — Supplementary material 1 (DOCX 94 kb) [file 426_2019_1278_MOESM1_ESM.docx]

**Supplemental Materials**

**Figure 1**


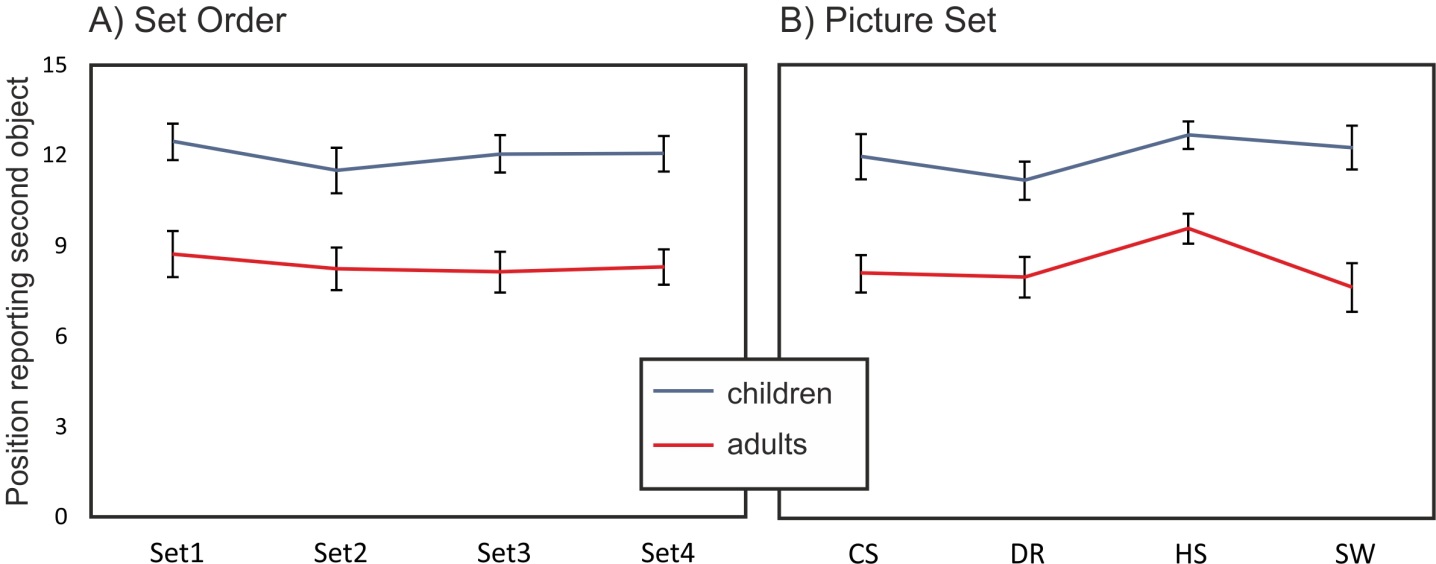


*Figure 1.* Average position at which children (blue line) and adults (red line) reported the second object, separately displayed for A. set-order and B. picture-set. CS = cat/swan, DR = duck/rabbit, HS = horse/seal, SW = snail/whale. Error bars reflect 95% confidence interval.
